# (Pro)renin Receptor Blockade Prevents Increases in Systolic Blood Pressure, Sodium Retention, and αENaC Protein Expression in the Kidney of 2K1C Goldblatt Mice

**DOI:** 10.3390/ijms26094177

**Published:** 2025-04-28

**Authors:** Pilar Cárdenas, Catalina Cid-Salinas, Allison C. León, Juan Castillo-Geraldo, Lilian Caroline Gonçalves de Oliveira, Rodrigo Yokota, Zoe Vallotton, Dulce Elena Casarini, Minolfa C. Prieto, Ramón A. Lorca, Alexis A. Gonzalez

**Affiliations:** 1 Instituto de Química, Pontificia Universidad Católica de Valparaíso, Valparaíso 2340000, Chile; pilar.cardenas@pucv.cl (P.C.); catalina.cid.s@mail.pucv.cl (C.C.-S.); allison.leon.l@mail.pucv.cl (A.C.L.); juan.castillo.g@mail.pucv.cl (J.C.-G.); 2 Departamento de Medicina, Disciplina de Nefrologia, Escola Paulista de Medicina, Universidade Federal de São Paulo, São Paulo 04023-062, Brazil; lilian.oliveira@unifesp.br (L.C.G.d.O.); yokotarodrigo@gmail.com (R.Y.); casarini.elena@unifesp.br (D.E.C.); 3 Department of Physiology and Tulane Hypertension and Renal Center of Excellence, Tulane University School of Medicine, New Orleans, LA 70112, USA; zvallotton@tulane.edu (Z.V.); mprieto@tulane.edu (M.C.P.); 4 Division of Reproductive Sciences, Department of Obstetrics and Gynecology, University of Colorado Anschutz Medical Campus, Aurora, CO 80045, USA; ramon.lorca@cuanschutz.edu

**Keywords:** renin, collecting duct, hypertension, PRO20, ProRENIN, renovascular hypertension

## Abstract

Physiological control of blood pressure (BP) and extracellular fluid volume is mediated by the action of the renin-angiotensin system (RAS). The presence of RAS components throughout the nephron has been widely discussed. The (pro)renin receptor (PRR) is a member of the RAS widely expressed in the body of humans and rodents. In the kidney, PRR is expressed in mesangial cells, renal vasculature, and tubules of the proximal and distal nephron. Binding of the PRR to renin and prorenin promotes angiotensin (Ang) I-mediated sodium (Na^+^) reabsorption via the epithelial sodium channel (ENaC). The Goldblatt 2-kidney 1-clip (2K1C) is a model of experimental renovascular hypertension that displays activation of systemic and intrarenal RAS. We use the 2K1C hypertension mouse model for 7 days to evaluate the role of the PRR on renal αENaC expression, Na^+^ reabsorption, and BP using pharmacological systemic blockade of the PRR with PRO20 peptide. Mice undergoing or not to 2K1C surgery (0.13 mm clip internal gap) were chronically infused with PRO20 and compared to sham (control) mice to assess changes in systolic BP (SBP) and diastolic BP (DBP), intrarenal angiotensin-converting enzyme (ACE) activity, Ang II, and renal αENaC expression and natriuretic responses after a saline challenge. Renal artery obstruction increased SBP and DBP, intrarenal ACE activity, Ang II levels, Na^+^ retention, and αENaC expression in both kidneys. PRO20 prevented the increases in SBP, DBP, attenuated Na^+^ retention, and blunted intrarenal Ang II and αENaC levels in non-clipped kidneys of 2K1C mice. Chronic infusion of the PRR blocker PRO20 for 7 days prevents hypertensive responses in part due to impaired αENaC upregulation and intrarenal Ang II formation in the early phase of the development of renovascular hypertension in 2K1C Goldblatt mice.

## 1. Introduction

Physiological control of arterial blood pressure and extracellular fluid volume is mediated by the renin-angiotensin system (RAS) [1]. Inappropriate activation of the systemic RAS markedly contributed to the development and progression of hypertension [2]. For this reason, the RAS is the main therapeutic target in treating hypertension [3]. Pharmacological treatments are mainly based on inhibitors of Ang II formation, i.e., inhibitors of angiotensin-converting enzyme (ACE) and blockade of angiotensin II (Ang II) receptors (AT1R) [4]. The RAS is a complex multi-organ hormonal system controlled by renin produced in the juxtaglomerular cells in the kidney in response to a decrease in renal perfusion [1]. Renin is released into the circulation, acting on the circulating angiotensinogen (AGT) produced by the liver to generate angiotensin I (Ang I). Ang I is then converted to Ang II by the ACE, which is abundantly expressed in the lungs and kidney tubules [5]. Ang II is the final effector of the RAS, stimulating vasoconstriction, aldosterone-mediated sodium (Na^+^) reabsorption via activation of epithelial Na^+^ channel (ENaC), and driving extensive distal Na^+^ reabsorption [6].

It has been shown that the intrarenal RAS is overactivated in different pathological conditions [7,8]. AGT is augmented in urine samples of patients with diabetic nephropathy, reflecting intrarenal RAS activation [9,10]. Tubular AGT serves as the substrate for renin, which is expressed in the principal cells of the collecting duct [11]. Prorenin (proenzyme) is also abundant in the principal cells of the collecting duct, and renin activity can be detected in intratubular fluids and urine samples [12]. Renin catalyzes the conversion of AGT to Ang I (biologically inactive), which is rapidly converted to Ang II by the ACE abundantly expressed in the collecting duct [13]. Importantly, the upregulation of tubular RAS components in hypertensive animal models [14,15,16] and urine samples from hypertensive patients [12,17] supports the role of the intratubular RAS in the development of hypertension. Indeed, intratubular concentrations of Ang II are greater than plasma [18,19]. Moreover, intrarenal Ang II generation is required for the development of hypertension [8] via the actions on AT1R in the distal nephron [20], activating ENaC and promoting Na^+^ transport in the collecting duct [21]. We demonstrated that Ang II increases renin expression and secretion in the principal cells of the collecting duct [11,22,23]. These effects oppose the mechanisms in juxtaglomerular cells, in which Ang II, via AT1R, suppresses the expression of renin as part of the negative feedback of Ang II [22]. Then, augmented intratubular Ang II contributes to the upregulation of prorenin and renin in collecting duct cells.

Recently, the (pro)renin receptor (PRR) has emerged as a key regulator of renal tubular Na^+^ reabsorption [24,25,26]. PRR binds prorenin or renin secreted by the neighboring principal collecting duct cell, fully activating prorenin and increasing renin catalytic activity [27]. This suggests that the upregulation of the PRR in the collecting duct may result in further intra-tubular Ang II formation [26,28,29]. Previous reports from our group showed that PRR interacts with prorenin and renin in the tubular fluid [30]. Then, it is expected that increases in both renin and PRR in the collecting duct may boost distal intratubular Ang II formation.

The 2-kidney, 1-clip (2K1C) renovascular hypertension model is widely used [31,32,33,34]. In this model, obstruction of the renal artery promotes the release of renin into the bloodstream and the activation of systemic RAS [35]. An increase in the PRR levels has been reported in 2K1C in rats [36]. Recently, we reported the induction of the PRR in mice subjected to 2K1C surgery for 14 days [35]. Therefore, an increase in the expression of the PRR in the 2K1C model may contribute to the increase in Ang II formation and ENaC activity. However, the contribution of the PRR on ENaC expression in both clipped and non-clipped kidneys in the 2K1C model during the early establishment of hypertension and its effect on Na^+^ balance has not been studied.

Several studies have used PRO20 [37,38,39], a 20 amino acid peptide that functions as a pharmacological inhibitor of the PRR (L1PTDTASFGRILLKKMPSVR). This peptide blocks the binding of prorenin, and it has been suggested to suppress the activation of intratubular RAS [38] and is a protective agent in renal failure [40,41]. In this report, we aimed to test if the pharmacological blockade of the PRR attenuates sodium retention and the increase in blood pressure associated with changes in the expression of the αENaC in the kidneys of mice with the model 2K1C.

## 2. Results

### 2.1. K1C Goldblatt Model Showed Increased Blood Pressure and Reduced Natriuretic Ability, However, PRO20 Prevented This Effect

**Figure 1:**
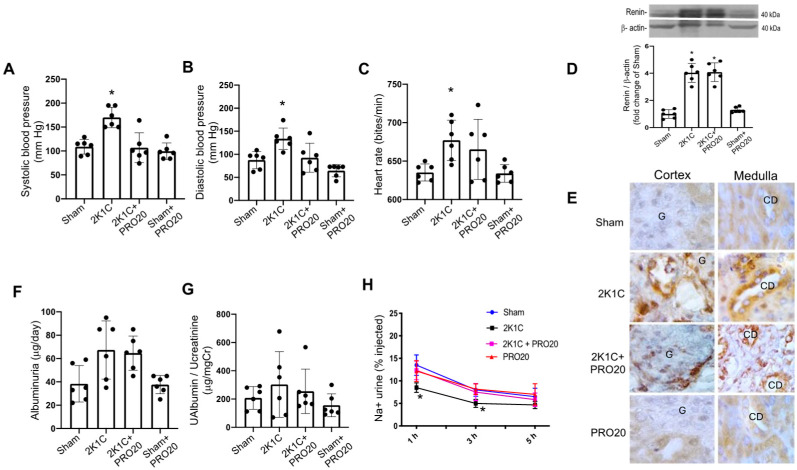
Physiological parameters and renin expression in mice subjected to 2K1C surgery with or without PRO20. Systolic (**A**) and diastolic (**B**) blood pressure were increased in 2K1C mice as compared to sham (controls); however, PRO20 was able to prevent this effect. Heart rate showed significant increases in 2K1C mice but not in 2K1C + PRO20 group (**C**). On day 7, urine collections were used to measure albuminuria and albumin/creatinine ratio. Increased juxtaglomerular renin expression evaluated by cortical tissues and immunoblot showed increased protein levels in 2K1C and 2K1C + PRO20 groups (**D**). Renin immunostaining was also augmented in both groups (**E**). There was a slight, but not significant increase in albuminuria in 2K1C and 2K1C + PRO20 mice (**F**). This trend was not observed when urinary albumin was corrected by urinary creatinine (**G**). Mice were subjected to a saline load challenge to test the ability of the mice to excrete a saline load. The sodium excretion was significantly lower in 2K1C group at 1 and 3 h post injection as compared with sham (controls). Mice subjected to 2K1C + PRO20 did not show Na^+^ retention (red line and squares). A saline test was performed on day 6 (**H**). * *p* < 0.05 vs. sham (controls). In Figure (**E**): G: Glomeruli; CD: collecting duct.

Under inhaled isoflurane anesthesia, the male of the C57BL/6J background (12-week-old male) underwent 2K1K Goldblatt surgery to place a silver clip with an internal diameter of 0.13 mm around the left artery. After one day of recovery, the mice were metabolically assessed and subjected to a protocol of 7 days with measurements of water intake, urine excretion, and food intake. No significant changes were found in water intake, urine excretion, or food intake in 2K1C or 2K1C + PRO20 groups. The systolic and diastolic blood pressure were measured in all groups using a tail-cuff sphygmomanometer. A shown in Figure 1, systolic and diastolic blood pressure were augmented in 2K1C mice; however, 2K1C mice chronically infused with PRO20 did not show increases in systolic or diastolic blood pressure (Figure 1A,B). Also, the heart rate was not changed in 2K1C mice treated with PRO20 (Figure 1C). Validation of the model was confirmed by the augmentation of cortical (juxtaglomerular) renin (Figure 1D). Immunostaining using an anti-prorenin/renin antibody showed increased levels in the cortex and medulla in 2K1C mice and 2K1C mice infused with PRO20 (Figure 1E). To check the presence and evidence of kidney damage in this model of 7 days, we evaluate albuminuria and albumin/creatinine ratio. As shown in Figure 1F,G, none of them were significantly altered in this model. A slight increase (not statistically significant) was seen in albuminuria (Figure 1F). Finally, we evaluated the natriuretic capacity in mice subjected or not subjected to 2K1C with or without PRO20. Mice were injected I.P. with a volume of warmed isotonic saline equivalent to 10% of their body weight and placed immediately in metabolic cages for urine collection. As shown in Figure 1H, 2K1C mice showed a decreased ability to excrete the injected Na^+^. This was observed at 1- and 3 h post-injection. Importantly, PRO20 did not show differences compared to controls, indicating that PRO20 prevents sodium retention.

### 2.2. Kidney Histological Characteristics

**Figure 2:**
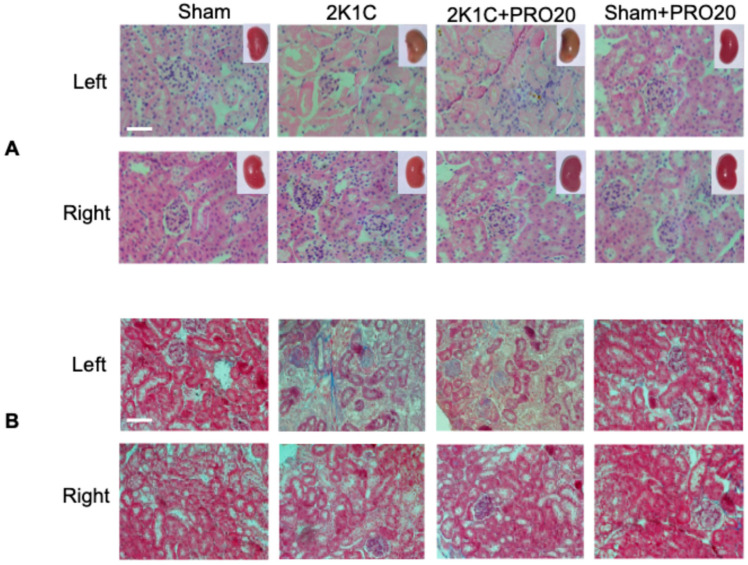
Kidney histological characteristics. (**A**) Hematoxylin and eosin staining of the left (clipped) and right (non-clipped kidneys) of sham-operated mice, 2K1C mice, 2K1C + PRO20 mice, and mice infused with PRO20. We did not observe changes in kidney size or weight. However, the color in the clipped kidneys was altered, as observed in the inserts. Left kidneys from 2K1C showed less preserved structures; in some areas, we observed loss of glomeruli structures and the presence of hyaline substances along with augmented glomerular size. The right kidneys in 2K1C and 2K1C + PRO20 groups showed enlarged glomeruli size and capillary to Bowman capsule distance. (**B**) Masson’s trichrome staining showed no evidence of fibrosis in the right kidneys subjected to 2K1C or 2K1C + PRO20 treatments; however, the left kidneys (clipped) showed an increase in blue staining intensity. Scale bar: 50 μm.

Compared to sham-operated kidneys, clipped kidneys from 2K1C mice did not show changes in kidney size or weight; the color in clipped kidneys was altered as observed in inserts of Figure 2A. When compared to Sham-operated kidneys, left kidneys from 2K1C showed less preserved structures, including loss of glomerular structures. Sham-operated kidneys, left kidneys from 2K1C, showed less preserved structures, including loss of glomerular structures. We also observed the presence of hyaline substances and augmented glomerular size (Figure 2A). Right kidneys in 2K1C and 2K1C + PRO20 groups showed enlarged glomeruli size (2K1C: 50 ± 6; 2K1C + PRO20: 48 ± 8 vs. control [sham]: 43 ± 4 μm, *p* < 0.05) and capillary to Bowman capsule distance (2K1C: 6 ± 1; 2K1C + PRO20: 7 ± 2 vs. control [sham]: 4 ± 1 μm, *p* < 0.05). Masson’s trichrome staining showed no evidence of fibrosis in the right kidneys subjected to 2K1C or 2K1C + PRO20 treatments; however, the left kidneys (clipped) showed an increase in blue staining intensity (Figure 2B) that was partially prevented by PRO20 treatment (Percentage of intensity: control [sham]: 12 ± 6%; 2K1C: 63 ± 11%, *p* < 0.001 vs. control [sham]; 2K1C + PRO20: 45 ± 12%, *p* < 0.05 vs. control [sham]; PRO20: 15 ± 8%, non-significant vs. control).

### 2.3. Intrarenal ACE Activity and Ang II Levels

**Figure 3:**
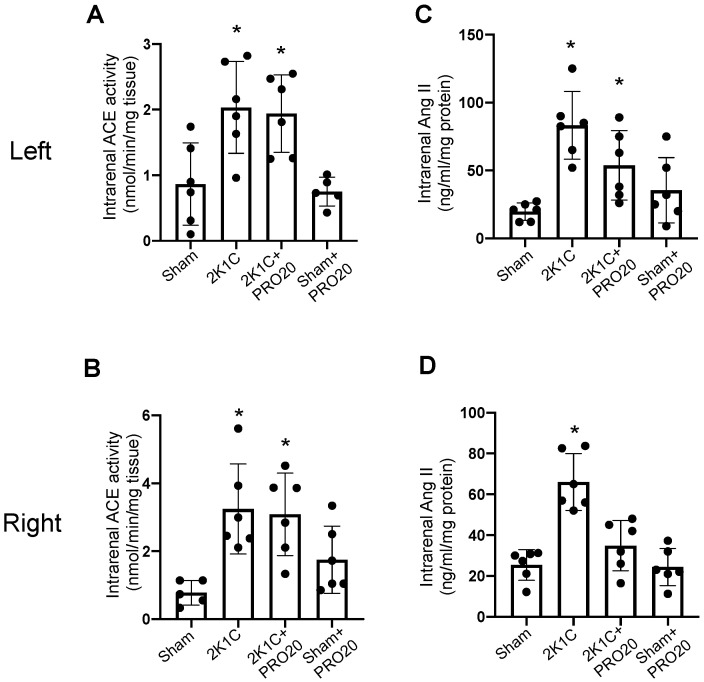
Intrarenal angiotensin-converting enzyme activity and Ang II levels. After 7 days of left artery clipping, the kidneys were extracted and intrarenal ACE activity and Ang II levels were measured in mice subjected to sham surgery (controls), 2K1C surgery, 2K1C + PRO20 (subcutaneous systemic infusions, and PRO20 treatment alone. Intrarenal ACE activity was augmented in clipped (**A**) and non-clipped (**B**) kidneys in 2K1C and 2K1C + PRO20 groups. Intrarenal Ang II levels increased in 2K1C and 2K1C + PRO20 groups in the clipped kidneys (**C**). By contrast, the augmentation of intrarenal Ang II in right kidneys (non-clipped) was prevented by PRO20 (**D**). * *p* < 0.05 versus sham (control) mice.

We next tested the ACE activity and intrarenal levels of Ang II as an indicator of the intrarenal RAS function. Intrarenal ACE activity was increased in the left (Figure 3A) and right (Figure 3B) kidneys from 2K1C mice subjected to 7 days of left renal arterial clipping. Infusion of PRO20 in 2K1C mice did not alter this effect. PRO20 alone did not show effects as compared to the controls. Interestingly, intrarenal Ang II was greatly augmented in the left and right kidneys in 2K1C mice; however, PRO20 partially (but not significantly) impaired this effect in the left and right kidneys in 2K1C mice. However, PRO20 partially (but not significantly) impaired this effect in the left kidney. In the right kidneys of 2K1C + PRO20, augmentation of Ang II levels was completely prevented. No effects were observed in the group treated with PRO20 alone.

### 2.4. Upregulation of the PRR in 2K1C and 2K1C + PRO20 Mice

**Figure 4:**
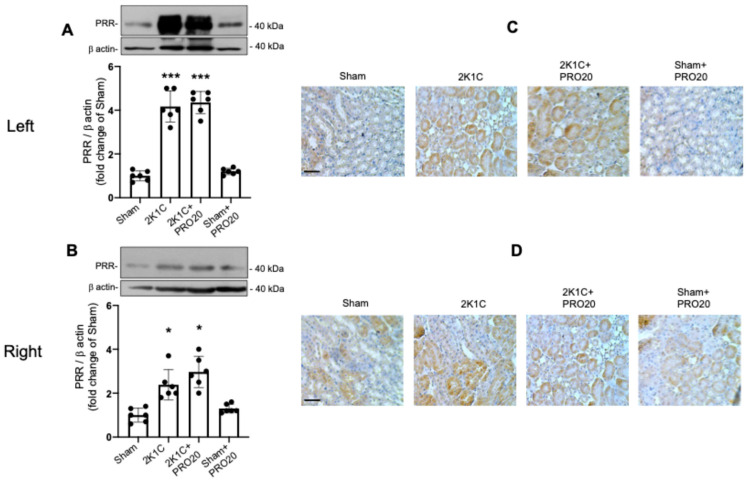
2K1C surgery increases PRR expression in clipped and non-clipped kidneys. We tested the effect of 2K1C surgery and 2K1C surgery plus PRO20 systemic infusions on PRR expression by semiquantitative immunoblotting and by immunostaining in kidney tissues. (**A**) A four-fold increase was observed in PRR protein expression in the clipped kidneys, while a similar effect was seen in mice with 2K1C surgery and PRO20 treatment. (**B**) Similarly, right kidneys showed a two-fold increase in PRR protein levels in the 2K1C and 2K1C groups. (**C**) Immunostaining of the PRR in renal tissues showed increased staining in 2K1C and 2K1C + PRO20 groups in the left (**C**) and right (**D**) kidneys. *** *p* < 0.001, * *p* < 0.05. Scale bar in (**C**,**D**): 50 μm.

Upregulation of the PRR has been reported in renovascular hypertension in rats [36]. PRR seems to be functionally relevant in the establishment of hypertension responses as part of intrarenal RAS [25,42,43]. Then, we evaluated protein expression in the clipped and non-clipped kidneys by immunoblot and immunohistochemistry. Protein levels of the PRR were augmented in left kidneys (fold change in control sham: 4.1 ± 0.2 vs. 1.0 ± 0.1, *p* < 0.001) and around two-fold in right kidneys (fold change in control [sham]: 2.3 ± 0.2 vs. 1.0 ± 0.1, *p* < 0.05). PRO20 did not have any effect on PRR increases in the left or right kidneys (Figure 4A,B). Immunostaining showed an increase in the intensity of brown-labeling in kidney tubules (mostly proximal and collecting ducts, as judged by tubular structures) in 2K1C and 2K1C + PRO20 groups (Figure 4C). Similarly, immunostaining in the right kidneys showed increased labeling in the 2K1C and 2K1C + PRO20 groups. No changes were observed in the PRO20 group.

### 2.5. Upregulation of αENaC in the Non-Clipped Kidneys of 2K1C Mice Is Prevented by PRO20

**Figure 5:**
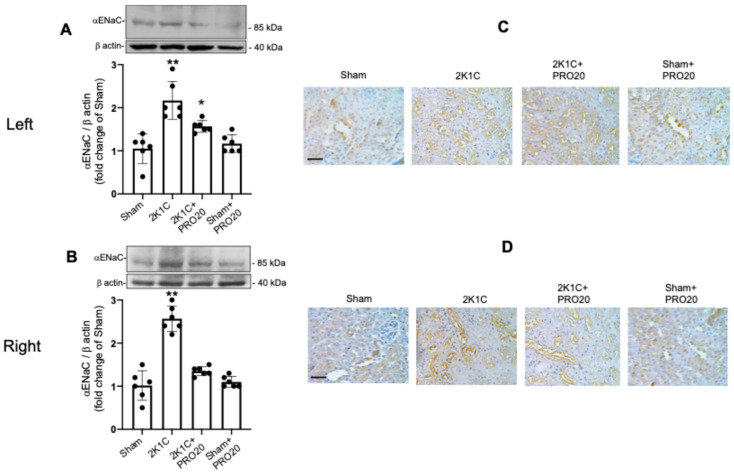
2K1C surgery increases αENaC expression in clipped and non-clipped kidneys, while PRO20 prevents this effect. Since PRO20 was able to prevent intrarenal Ang II levels in both kidneys, and because Ang II may be able to regulate ENaC abundance, we evaluated the expression of αENaC subunit. The 2K1C surgery increased αENaC expression in the clipped kidney that was partially prevented by PRO20 (**A**). In contrast, PRO20 systemic infusions in 2K1C mice completely blunted αENaC subunit upregulation after 7 days (**B**). Immunostaining analysis in the left (**C**) and right (**D**) kidneys confirmed the evidence demonstrated by immunoblot. ** *p* < 0.01, * *p* < 0.05 versus the sham (control) group. Scale bar in (**C**,**D**): 50 μm.

Since we observed a more efficient natriuretic response after the sodium challenge in 2K1C mice infused chronically with PRO20 on day 6 compared with 2K1C mice, we evaluated if this effect was related to changes in ENaC protein expression, particularly αENaC subunit. Since the increased levels of intrarenal Ang II were prevented in 2K1C + PRO20 group, and ENaC can be regulated directly by Ang II in tubular segments 45, we tested the protein abundance of αENaC by immunoblotting and immunostaining. The left kidneys of 2K1C mice showed increased abundance of αENaC (fold change in control [sham]: 2.2 ± 0.2 vs. 1.0 ± 0.1, *p* < 0.01, Figure 5A) as well as in right kidneys (fold change in control: 2.6 ± 0.1 vs. 1.0 ± 0.1, *p* < 0.01, Figure 5B). Interestingly, the increases in protein abundance of αENaC were only partially prevented in the left kidneys of mice subjected to 2K1C surgery and systemically infused with PRO20 for 7 days (fold change in control [sham]: 1.5 ± 0.1 vs. 1.0 ± 0.1, *p* < 0.05, Figure 5A); however, in the right kidneys PRO20 treatment blunted the increased abundance of αENaC observed in kidneys from 2K1C mice (fold change in control [sham]: 1.2 ± 0.1 vs. 1.0 ± 0.1, *p* < 0.05, Figure 5B). Representative immunostaining of αENaC in the left (Figure 5C) and right (Figure 5D) confirmed the results seen by immunoblotting.

## 3. Discussion

In the present study, we demonstrated that during the early phase of the two-kidney, one-clip (2K1C) Goldblatt renovascular experimental hypertensive mouse model, there is an increased expression of PRR protein abundance along with augmented αENaC levels in the clipped kidney and non-clipped kidney. In this phase of early hypertensive response, the animals exhibit around 50 mm Hg increases in systolic blood pressure, which is similar to what has been previously observed [34,35,44]. We also described the augmented activity of ACE in both clipped and non-clipped kidneys (Figure 3). We also observed an increased intrarenal Ang II in clipped and non-clipped kidneys (Figure 3). We were able to confirm that this model can increase juxtaglomerular renin levels (Figure 1D,E), thus increasing systemic RAS. As described previously in rats [36,45], prorenin/renin staining in renal collecting ducts was also increased in 2K1C mice. Although we found a slight increase in albuminuria at day 7, the analysis of urinary albumin vs. urinary creatinine did not show significant differences when comparing 2K1C mice versus sham controls. In addition, the evaluation of histological aspects showed a slight increase in kidney damage and altered renal structures, such as glomerular size and tubular alterations in the clipped kidney. However, we did not observe alterations in the non-clipped kidney (Figure 2).

Once the model was validated, we tested the effect of systemic infusions of PRO20, a 20-amino acid peptide that functions as a pharmacological inhibitor of the PRR that blocks the binding of renin/prorenin, which has been used in several studies [38,46,47,48]. The left renal clipping after 7 days caused an increase in systolic blood pressure, renal Ang II, ACE activity, and augmented αENaC protein levels, all of which were suppressed by co-administration of PRO20 through osmotic subcutaneous mini-pumps. Indeed, several studies have shown that intrarenal RAS components are augmented in the 2K1C model. For instance, ACE activity is augmented in 2K1C rats [49]. Moreover, it has been shown that angiotensinogen expression and urinary angiotensinogen excretion are observed in the nonclipped kidneys of 2K1C hypertensive rats [50].

The PRR plays an important role in the activation of the tubular RAS [51]. Our group and others have demonstrated that ablation of the PRR in the collecting duct blunted the responses to increased blood pressure during chronic infusions of Ang II [26,52]. Also, the specific nephron-specific deletion of the PRR causes a urine concentration defect [25]. Moreover, the absence of the PRR in the collecting ducts impairs intrarenal Ang II formation and increases urinary renin activity during experimental hypertension [26], suggesting that PRR binding to prorenin or renin enhances intratubular Ang II, promoting Na^+^ reabsorption, impacting blood pressure. Our data are consistent with the fact that PRR blockade was able to prevent the development of hypertension in 2K1C mice. Krebs et al. showed that in hypertensive Goldblatt rats, the PRR was upregulated in the clipped kidney. Immunohistochemistry studies also showed a redistribution of renin upstream from the glomerulus in preglomerular vessels and renin staining in tubular cells. The authors also showed that the expression of the PRR was increased, particularly in renal tubules [53]. This is consistent with the fact that both prorenin/renin and PRR in the collecting ducts are augmented, supporting the overactivation of intratubular RAS [49].

The PRR also exists as a soluble form that is cleaved by Furin [54]. Wang et al. showed the effect of the soluble PRR (sPRR) on αENaC regulation and its role in aldosterone signaling. They used cultured mpkCCD cells treated with histidine-tagged sPRR, showing the induction of amiloride-sensitive currents. They also demonstrated that primary rat inner medullary collecting duct cells, treated with sPRR, were able to induce the expression of the αENaC. Moreover, by examining short-circuit current in Ussing chambers, they showed that aldosterone-induced transepithelial Na^+^ transport was inhibited by the PRO20. This evidence also supports our results and demonstrates that PRR and sPRR induce ENaC activation and function as regulators of the aldosterone pathway [55].

Most of the evidence using the 2K1C model has been studied in rats and mice in periods of two or four weeks, in which PRR upregulation can be associated with the thickening of renin-positive vessels and tubulointerstitial damage, suggesting that PRR may play a profibrotic role in the clipped kidneys of Goldblatt rats [53]. The involvement of activated prorenin in the pathogenesis of slowly progressive nephropathy in the non-clipped kidney of two-kidney, one-clip hypertension has also been proposed. Ryuzaky et al. showed that in the non-clipped kidneys at 12 weeks after 2K1C surgery, activated prorenin levels, Ang II levels, and transforming growth factor (TGF)-β mRNA levels of HRP-treated rats were prevented as compared to 2K1C rats, suggesting again that PRR-dependent activation of prorenin contributes to the pathogenesis of slowly progressive nephropathy in the contralateral kidney in a rat model of renovascular hypertension [56]. Despite this evidence, other articles have shown that the PRR peptide inhibitor, the handle-region peptide, does not affect hypertensive nephrosclerosis in Goldblatt rats [57]. In our model of 7 days of clipping, we did not observe albuminuria and no changes as compared to the controls or even with co-treatment with PRO20, despite the increases in blood pressure.

Regarding Na^+^ handling in the 2K1C model, we have shown that 14 days after clipping, 2K1C mice show decreased natriuretic responses [35]. We observed similar responses on day 6. In a chronic setting, Ang II treatment induced αENaC mRNA expression in mpkCCD cells, which was similarly blocked by PRO20 [39]. Also, chronic intramedullary infusion of an ENaC inhibitor, amiloride, in rats significantly attenuated Ang II-induced hypertension [39]. Overall, the present study suggests that CD PRR contributes to Ang II-induced hypertension at least partially via activation of renal medullary ENaC. Correa et al. recently demonstrated the effect of dual RAS blockade on ENaC expression in rats subjected to 2K1C surgery. They observed that 2K1C rats presented lower levels of plasma potassium associated with higher fractional excretion of potassium and increased expression of the αENaC and γENaC. On the other hand, 2K1C rats treated with ACE inhibitors or Ang II receptor blockers had lower markers of ENaC activation. Importantly, dual RAS blockade decreased the renal cortical abundance of cleaved αENaC to levels that were even lower than in normotensive rats [58].

Limitations of the study: Several reports on different organs and systems support the role of the PRR in a variety of functions, and many of these roles are still being actively researched. Because we used a systemic infusion of PRO20 via subcutaneous minipumps, we cannot rule out the possibility that PRO20 may have actions in other organs such as the brain, heart, or adipose tissues, and even in immune cells. The activation of the PRR stimulates intracellular pathways related to cardiac damage [59]. Mahmud et al. quantified the expression of the PRR in several animal models of heart failure; in post-myocardial infarcted hearts, they found increased PRR mRNA levels. Moreover, they observed significant increases in PRR levels in the hearts of patients with dilated cardiomyopathy [60,61]. Adipose tissue synthesizes all of the components of the RAS [62], and it has been shown that the PRR increases ERK ½ signaling pathways [63]. Then, it is also reasonable that actions of systemic PRO20 may act on cardiac or adipose tissue RAS activation. Furthermore, since hypertensive responses are also related to immune actions [64,65] and the PRR is present in lymphocytes and monocytes, [66] shows that the actions of PRO20 may likely affect PRR function in immune cells.

Conclusions: Our study demonstrates that during the early phase of renovascular hypertension, there is an upregulation of renin synthesis in juxtaglomerular cells as early as day 7. This increase in juxtaglomerular renin reflects systemic RAS activation impacting blood pressure and natriuretic impairment in response to a saline challenge. This is concomitant with the upregulation of αENaC in the clipped and non-clipped kidneys, demonstrating that in this model of renovascular hypertension, both kidneys have active mechanisms of Na^+^ reabsorption. The observed increases in blood pressure and Na^+^ retention were also associated with augmented intrarenal ACE activity and Ang II levels, which reinforce previous evidence showing increased intrarenal RAS in this model. Along with the increased intrarenal RAS, the abundant expression of the PRR in the collecting duct and its upregulation by high levels of circulating Ang II suggested an interesting role in prorenin and renin activation, along with further intratubular Ang II formation. The strong evidence of the role of the PRR even in water and Na^+^ handling in the kidney suggests that PRR may be a potential target for the treatment of hypertension. In our study, we found that chronic systemic infusion of PRO20 was able to prevent high blood pressure, attenuating intrarenal Ang II levels and suppressing αENaC expression, which also parallels increased natriuretic responses. This indicates that renal PRR has an important role in the physiology of water and electrolyte homeostasis, despite possible actions in the rest of the body. As mentioned before, we cannot discard the possibility that the actions of PRO20 may also be related to the blockade or PRR in other target organs. It seems that PRR-dependent Ang II formation linked to Na^+^ reabsorption strongly impacts blood pressure. We emphasize the potential impact that this type of research may have on the study of the mechanisms involved in the regulation of vasoactive peptides along the nephron and pathologies such as diabetes, ischemic nephropathy, and renal fibrosis, among others. We believe that targeting the intrarenal or systemic PRR may open future studies to therapeutic approaches for the treatment of hypertension.

## 4. Materials and Methods

### 4.1. Animals

Mice of C57BL/6J background (12-week-old male) were used for the studies by the Declaration of Helsinki and bioethical protocol described in accordance with the Institutional Review Board from the Pontificia Universidad Católica de Valparaíso (protocol code BIOEPUCV-BA 482-2022). Mice were divided according to the specific protocol using *n* = 6 animals per group: Sham-operated (sham); 2K1C surgery (2K1C), 2K1C surgery plus minipump implantation with PRO20 (700 μg/Kg/day); (2K1C + PRO20) and PRO20 (700 μg/Kg/day) alone.

#### K1C Goldblatt Surgery

The left renal artery was exposed via a left flank incision and isolated carefully. A silver clip with an internal diameter of 0.13 mm was placed around the left artery to reduce whole-kidney blood flow in the clipped kidney [32,34]. A flank incision without clamping was used in the control (sham) mice. After the surgery, the skin wound was cleaned with Betadine and closed with surgical clips. Mice were monitored in cages until they reached full recovery. The effect of blood flow reduction was confirmed by erythropoietin expression and changes in the color of the kidney.

### 4.2. PRO20 Peptide Administration

On day 0 (with or without 2K1C surgery), mice were lightly anesthetized with 2.5% isoflurane delivered in a box. PRO20 was administered at a dosage of 700 μg/Kg/day via osmotic minipumps for 7 days at a delivery rate of 0.5 μL/h (ALZET Model 2001, Cupertino, CA, USA) containing a vehicle of either 200 μL of saline or PRO20. Montelukast was dissolved in 200 µL of saline. Osmotic minipumps were implanted under the back skin through a small skin incision under isoflurane anesthesia.

### 4.3. Blood Pressure Measurements

Systolic and diastolic arterial blood pressure was measured in all groups by using a tail-cuff sphygmomanometer. The equipment (CODA system, Kent Scientific, Torrington, CT 06790, USA) allowed multifunctional monitoring capability (systolic, diastolic, and mean blood pressure as well as heart rate). The chamber was warmed at 36 °C and mice were trained for 5 days. The measurements were performed on day 6 of treatment before euthanasia.

### 4.4. Urine Flow, Water Intake, Food Intake, and 24-Hour Sodium Excretion

Mice were placed in conditions of the light–dark cycle (12 h), temperature of 21 °C, humidity of 50%, adequate ventilation, noise free, food and water ad libitum during the protocols. On the day before the beginning of the protocol and days 10 and 13, animals were placed in metabolic cages (Rotarod, Ugo Basile, Washington, DC, USA) for 24 h for data collection of urine flow, water, and food intake. Animals were monitored every 3 to 6 h to check their health status during the whole protocol.

### 4.5. Saline Challenge Test

A saline challenge was performed on day 6 to evaluate the natriuretic capacity. Mice were injected I.P. with a volume of warmed isotonic saline equivalent to 10% of their body weight and placed immediately in metabolic cages for urine collection. Results are expressed as the percentage of the injected sodium excreted over 5 h.

### 4.6. Immunoblotting Analyses

Forty micrograms of protein samples were electrophoretically separated on a precast NuPAGE 10% Bis-Tris gel (Novex) at 200 volts for 45 min followed by semi-dry transference to a nitrocellulose membrane (Invitrogen) using iBlot (Invitrogen, Carlsbad, CA, USA). Blots were blocked at RT for 3 h, incubated overnight with specific primary antibody at 4 °C, incubated with the corresponding secondary antibodies (1:5000 dilutions), at room temperature for 45 min and then analyzed by normalization against β-actin, used as a housekeeping gene. PRR protein levels were detected using a polyclonal rabbit anti-PRR (ATP6AP2, 1:200; Cat. HPA003156, Sigma-Aldrich, St. Louis, MO, USA) that recognizes the intracellular segment and the ectodomain. The alpha subunit of ENaC was detected by using polyclonal rabbit anti SCNN1A (Cat. LS-C150448-100, LS Bio, Newark, CA, USA) Tissue immunoblots are presented in each figure as representative images for each experimental group corresponding to *n* = 6. Results are presented as the ratio of analyzed protein versus β-actin (Cat. A1978, Sigma-Aldrich), and presented as fold change in the control.

### 4.7. Histology

Sagittal sections of kidneys were fixed in Bouin (Sigma Aldrich, St. Louis, MO, USA), and embedded in paraffin. We prepared 5 μm sections using a Leica RM2235 microtome (Leica Microsystems, Shanghai, China). The sections were washed and stained with an hematoxylin–eosin stain (Sigma Aldrich, St. Louis, MO, USA). Images were captured with a Nikon digital sight DS-U3 digital camera, attached to a Nikon Upright Microscope ECLIPSE Ci-L (Nikon Instruments Inc., Melville, NY, USA), and analyzed using ImageJ 1.43u software (NIH, USA). Masson’s trichrome staining was performed using Trichrome Stain (Masson) Kit (ABCAM, Cambridge, UK). Ten representative fields were subjected to measurements of blue staining (collagen) intensity and expressed as a percentage of intensity as compared to control.

### 4.8. Immunostaining of Renin, PRR and αENaC

Kidney slides 3 μm were fixed and stained with anti-PRR at 1:200 dilutions (ATP6AP2, Cat. #HPA003156, Sigma-Aldrich, St. Louis, MO, USA), anti αENaC at 1:100 dilutions (Cat. # PAI-920A, Invitrogen, CA, USA). The images were obtained using a Nikon Eclipse-50i microscope (Nikon Instruments Inc., Melville, NY, USA) and were digitized using the NIS-Elements BR version 4.0 from Nikon). Negative controls were obtained by omission of the specific primary antibody.

### 4.9. Angiotensin II Kidney Content

Renal levels of ANG II were measured in kidney sample homogenates enriched in medullary tissues using ELISA Kit for Ang II (Cloud-Clone Corp, Katy, TX, USA) according to the manufacturer’s instructions.

### 4.10. ACE Enzymatic Activity

ACE catalytic activity was determined fluorometrically as described by Friedland and Silverstein [67] modified by Ronchi et al. [68]. The freeze-dried samples were resuspended in 1 mL of 100 mM sodium borohydride buffer, pH 7.2, containing 340 mM sucrose and 300 mM NaCl, and a protease inhibitor cocktail (complete mini EDTA-free; Roche, St Albans AL1 3EW Hertfordshire, UK) was added. The protein concentration was determined using the Bio-Rad Protein Assay Dye Reagent Concentrate (Cat. no. #500-0006, Bio-Rad, Hercules, CA, USA), according to the manufacturer’s instructions. ACE activity was determined by a fluorometric method using Z-Phe-His-Leu-OH as substrate. Briefly, 10 µL of the homogenate sample was incubated with the substrate diluted in borate buffer containing ZnSO_4_ at 37 °C for 10 min. Enzymatic activity was interrupted by 0.28 M NaOH. The dipeptide His-Leu released by cleavage reacted with orthophthalaldehyde forming a fluorescent adduct. The reaction was stopped with the addition of 3 M HCl after 10 min. Fluorescence intensity was measured using a spectrofluorometer (Infinite^®^ 200 Pro, Tecan, Männedorf, Switzerland) with excitation at 360 nm and emission at 465 nm. ACE activity was corrected by protein concentration and expressed as nmol/min/mg of protein.

### 4.11. Urinary Creatinine

Urinary creatinine was measured using Quidel Kit according to the manufacturer’s guide (San Diego, CA, USA). Urine samples and standards were diluted to 1:40, and 25 µL was added to a 96-well plate plus 75 µL of colored solution. The reaction was stopped using 2 N H_2_SO_4_ and the absorbance was read at 490 nm.

### 4.12. Urinary Albumin

Urinary albumin was measured by mouse albumin ELISA kit (Catalog # EEL119, Invitrogen, Carsbad, CA, USA) according to the manufacturer’s instructions.

### 4.13. Statistical Analysis

The results are expressed as mean ± SEM. Statistical analyses were performed using GraphPad Prism Software Version 8 (GraphPad Software, Inc., La Jolla, CA, USA). The normal distribution of each parameter analyzed was tested using Shapiro–Wilk test. One-way ANOVA was used to compare the mean differences between groups. Post-test comparisons for two groups by non-paired (one-tailed) *t*-test were also used. A *p*-value < 0.05 was considered statistically significant.

## Data Availability

The authors will make all methods and materials, the experimental design to induce 2K1C Goldblatt hypertension, among others, available to other researchers, as requested.
